# Modified Si–Jun–Zi–Tang Attenuates Airway Inflammation in a Murine Model of Chronic Asthma by Inhibiting Teff Cells via the mTORC1 Pathway

**DOI:** 10.3389/fphar.2019.00161

**Published:** 2019-02-27

**Authors:** Hualiang Jin, Cui Cai, Bei Li, Weizhong Jin, Junbo Xia, Limin Wang, Shenglin Ma

**Affiliations:** ^1^Department of Respiratory Diseases, Affiliated Hangzhou First People’s Hospital, Nanjing Medical University, Hangzhou, China; ^2^Department of Respiratory Diseases, Affiliated Hangzhou First People’s Hospital, Zhejiang University School of Medicine, Hangzhou, China; ^3^Department of Geriatric Medicine, Red Cross Hospital, Hangzhou, China; ^4^Department of Geriatric Medicine, Affiliated Hangzhou First People’s Hospital, Zhejiang University School of Medicine, Hangzhou, China; ^5^Department of Oncology, Affiliated Hangzhou First People’s Hospital, Nanjing Medical University, Hangzhou, China

**Keywords:** modified Si–Jun–Zi–Tang, asthma, airway inflammation, T effector lymphocytes, mTORC1

## Abstract

**Background:** Modified Si–Jun–Zi–Tang (MSJZT), a multi-herb formulation, is frequently used in traditional Chinese medicine for patients during the remission stage of asthma. However, the pharmacological basis underlying the effects of MSJZT on asthma has yet to be elucidated. This study aims at evaluating the anti-asthmatic effects of MSJZT and investigating its possible mechanism.

**Methods:** A chronic murine model of asthma was established by sensitization and repeated challenge with ovalbumin (OVA) in female BALB/c mice, followed with oral administration of MSJZT during remission, and then mouse were re-challenged by OVA. The chemical profile of MSJZT was analyzed by high-performance liquid chromatography. The characteristic features of allergic asthma, including airway hyperreactivity, histopathology, cytokine levels (IL-4, -5, -13, -17, and INF-γ), T regulatory (Treg) lymphocytes (Foxp3+CD4+CD25+), and T effector (Teff) lymphocytes (Foxp3-CD25+CD4+) in bronchoalveolar lavage fluid (BALF), and downstream proteins of mTORC1/2 signaling pathway were examined.

**Results:** MSJZT markedly suppressed airway hyper-responsiveness to aerosolized methacholine, and reduced levels of IL-4, IL-5, and IL-13 in the BALF. Histological studies showed that MSJZT significantly reduced inflammatory infiltration in lung tissues. The percentage and absolute number of Teff cells were suppressed to a remarkable level by MSJZT without affecting Treg cells. Furthermore, MSJZT effectively inhibited the mTORC1 activity, but exerted limited effects on mTORC2, as assessed by the phosphorylation of the mTORC1 and mTORC2 substrates, S6 ribosomal protein, p70 S6 kinase, mTOR S2481, and Akt, respectively.

**Conclusion:** MSJZT attenuated chronic airway inflammation in a mouse model of asthma by inhibiting Teff cells, which occurred, at least in part, via modulation of the mTORC1 signaling pathway.

## Introduction

Bronchial asthma is a heterogeneous chronic inflammatory disease characterized by airway inflammation, AHR, and structural airway remodeling (Saglani and Lloyd, 2015). Current clinical therapies, including glucocorticoids (GCs) and long-acting β2-adrenergic agonists, are widely used to control asthma. However, both the incidence and prevalence of asthma remain high (Gershon et al., 2010). Studies suggest that inhaled GCs, similarly to those administered systemically, can lower the immune response toward various pathogens, both within and outside of the respiratory system (Zuska-Prot and Maslanka, 2017). Therefore, it is imperative that the mechanisms underlying this disease are further identified, and new therapies developed.

CD4+ T cells play a crucial role in the pathogenesis of asthma and can be activated to differentiate into Teff lymphocytes. These cells, including Th2/Th17 cells, have been demonstrated to mediate lung inflammation and orchestrate pulmonary immune responses (Veres et al., 2017). Recently, another pivotal subset of CD4+ T lymphocytes, comprised of CD4+CD25+Foxp3+ Treg cells, has been increasingly recognized for its crucial role in the resolution of allergic airway disease (McGee and Agrawal, 2009). However, the mechanisms underlying how these CD4+ T cells are regulated in asthma have yet to be fully elucidated. mTOR, the mechanistic target of rapamycin, has been suggested to play a significant role in the differentiation and proliferation of Teff and Treg cells (Powell and Delgoffe, 2010). Suppression of mTOR by rapamycin can inhibit the proliferation of Th2 cells or induce expansion of the naturally occurring Treg cells *in vitro* (Powell et al., 2012). Furthermore, aberrant mTOR signaling is involved in the pathogenesis of asthma, and the inhibition of mTOR has been shown to attenuate key characteristics of allergic asthma, including airway inflammation, AHR, and goblet cell metaplasia (Mushaben et al., 2011). It is therefore clinically beneficial to screen new drugs for asthma that target mTOR signaling.

Modified Si–Jun–Zi–Tang, a traditional Chinese formula composed of *Astragalus membranaceus* (Fisch.) Bunge, *Atractylodes macrocephala Koidz., Citri Reticulatae Pericarpium, Codonopsis pilosula* (*Franch*.) *Nannf., Glycyrrhiza glabra L., Pinellia pedatisecta* Schott, and *Poria cocos* (*Schw.*) *Wolf*, has been frequently used in thousands of Chinese medicine prescriptions. A multicenter clinical study showed that MSJZT could exert therapeutic effect on childhood bronchial asthma with improving symptoms and lung function, and in the animal study part, MSJZT was demonstrated to inhibit airway infiltration of eosinophil and prevent the bronchoconstriction in an asthma model (Hsieh, 1996; Clark et al., 2010). However, the pharmacological basis underlying the effects of MSJZT on asthma still needs to be elucidated. Recently, *A. membranaceus* and *Glycyrrhiza*, key herbs in MSJZT, were showed to have inhibitory effects on the mTOR signaling pathway (Yo et al., 2009; Zhou et al., 2018). Therefore, the goal of this study was to confirm the anti-asthmatic effects of MSJZT during the remission phase in a murine model of established asthma, and to investigate the possible mechanisms of underlying the action of MSJZT, with particular emphasis on the mTOR signaling pathway.

## Materials and Methods

### Animals

Sixty female BALB/c mice (age 6 weeks), weighing 20–22 g, were purchased from Shanghai SLAC Laboratory Animal Co., Ltd., Shanghai, China. Animals were housed under pathogen-free conditions with food and water freely available in a temperature controlled environment (22 ± 2°C) on a 12-h light–dark schedule (lights on from 6:00 am to 6:00 pm). The protocol of the study was approved by the Committee on the Ethics of Animal Experiments of Zhejiang Chinese Medical University (permit number: ZSLL-2016-37; February 2016).

Mice were randomly divided into six groups (*n* = 10 per group) as follows: (1) a normal saline control (saline) group, (2) an OVA model group, (3) a dexamethasone (Dex) group (0.5 mg/kg), (4) a low dose MSJZT group (12.5 g/kg), (5) a median dose MSJZT group (25 g/kg), and (6) a high dose MSJZT group (50 g/kg).

### Reagents and Materials

Ovalbumin (Grade V) and Mch were purchased from Sigma–Aldrich China, Shanghai, China. Imject alum adjuvant, a formulation of aluminum hydroxide and magnesium hydroxide, was obtained from Thermo Fisher Scientific Co., Shanghai, China. Injectable Dex sodium phosphate was provided by Tianjin Pharmaceutical Group Xinzheng Co., Zhengzhou, China. ELISA kits for OVA specific IgE, INF-γ, IL-4, IL-5, IL-13, and IL-17A were purchased from Hangzhou Biological Pharmaceutical Co., Zhejiang, China. Antibodies for phospho-p70S6K (Thr389), p70 S6K, phospho-S6 ribosomal protein (Ser235/236), S6 ribosomal protein, phospho-Akt (Ser473), Akt, phospho-4E-BP1 (Ser65), 4E-BP1, phospho-mTOR (Ser2481), and mTOR were obtained from Cell Signaling Technology, Danvers, MA, United States.

### Drug Preparation

All herbs used in the study were commercially available dry materials, and were purchased from Zhejiang Yingte Pharmaceutical Co., Hangzhou City, Zhejiang, China. The preparation was a mixture of seven Chinese herbal medicines including *A. membranaceus* (*Fisch*) *Bunge* (36 g), *A. macrocephala Koidz* (36 g), *Citri Reticulatae Pericarpium* (21 g), *C. pilosula* (Franch.) Nannf (36 g), *G. glabra L.* (15 g), *P. cocos* (Schw.) *Wolf* (36 g), and *P. pedatisecta Schott* (21 g). This mixture was soaked in distilled water for 30 min and then extracted with 1.5 l of boiled water twice for 1 h. The decoction was then filtered through eight layers of gauze and the filtrate was vacuum evaporated to a final density of 2 g/ml at 65°C by a rotavapor. The final concentrations used for oral administration were equivalent to 12.5, 25, and 50 g of raw herb/kg body weight.

### High-Performance Liquid Chromatography (HPLC) Analysis

The chemical profile of MSJZT was analyzed by HPLC using methods described previously (Lu et al., 2016). HPLC analysis was performed using a Shimadzu LC-20AT system (Tokyo, Japan) equipped with an SPD-20A UV-detector and a CBM-102 workstation. The mobile phase consisted of solvent A (water with 0.1% phosphoric acid) and solvent B (acetonitrile). An elution program was performed as follows: 0–10 min at 10% of B, 10–20 min at 13% of B, 20–25 min at 17% of B, 25–27 min at 18% of B, 27–33 min at 21% of B, 33–38 min at 21% of B, 38–43 min at 24% of B, 43–63 min at 25% of B, 63–73 min at 45% of B, and 73 min at 80% of B. The flow rate was 1 ml/min, and the total injection volume was 10 μl. Data analysis was performed using the Similarity Evaluation System for Chromatographic Fingerprint of Traditional Chinese Medicine.

### Mouse Sensitization, Challenge, and Drug Administration

**FIGURE 1:**
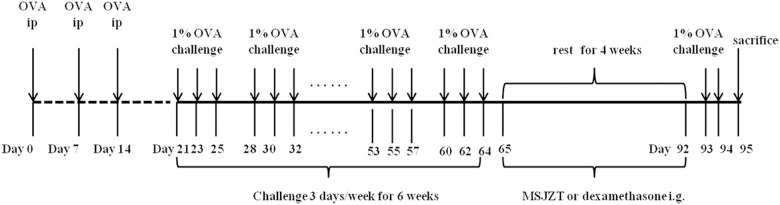
Experimental protocol. Mice were immunized three times with an intraperitoneal injection of a suspension containing 40 μg OVA (Grade V) and 0.05 ml alum adjuvant (a formulation of aluminum hydroxide and magnesium hydroxide). One week after immunization, mice were challenged with aerosolized OVA three times per week for 6 consecutive weeks, followed by 4 weeks of rest. Finally, mice were re-exposed to OVA challenge for 2 days. From days 65 to 92, mice in the treatment groups were given oral MSJZT at 12.5, 25, and 50 g raw herbs/kg body weight or 0.5 mg/kg/day dexamethasone (Dex).

As previously described, a murine model of asthma from chronic OVA sensitization and challenge was utilized (McMillan et al., 2005). In brief, mice were immunized at 0, 7, and 14 days by a peritoneal injection of 0.2 ml of sterile saline containing 40 μg of OVA and 0.05 ml of alum adjuvant. One week after sensitization, the mice were challenged with aerosolized OVA for 30 min/day from day 21. This OVA challenge was performed three times per week for 6 consecutive weeks, followed by 4 weeks of rest. From days 65 to 92, mice in the treatment groups were administered with MSJZT or Dex once per day via oral gavage, at doses of 12.5, 25, and 50 g raw herb/kg body weight for MSJZT and 0.5 mg/kg/day for Dex. Finally, mice were re-exposed to the OVA challenge for 2 days. Mice in the saline control group were challenged with the same volume of normal saline instead of OVA and received the same volume of normal saline for treatment. The protocol for sensitization, challenge, and drug administration is summarized in Figure 1.

### Measurement of AHR

Airway responsiveness to Mch was evaluated 24 h after the final OVA exposure using WBP with Buxco’s unrestrained WBP systems, as described previously (Pichavant et al., 2007). In brief, the mice were placed in a closed chamber, and the pressure fluctuations that occurred during the breathing cycle were recorded. After reaching a stable baseline the mice were given aerosolized PBS or various concentrations of Mch (6.25, 12.5, or 25 mg/ml) via the chamber and a jet nebulizer. A dimensionless parameter, referred to as “Penh,” was used as an indicator for changes in airway resistance (Finotto et al., 2001).

### Serum Collection and IgE Determination

Immediately following the assessment of AHR, blood samples were collected from all experimental animals after full anesthetization by intraperitoneal injection of pentobarbital sodium. After centrifugation at 3000 × *g* for 10 min, the serum was collected and stored at -80°C. OVA-specific IgE levels were then measured by ELISA in accordance with the manufacturer’s instructions. After serum collection, mice were euthanized by cervical dislocation.

### Preparation of Broncho-Alveolar Lavage Fluid (BALF) and Cytokine Analysis

Broncho-alveolar lavage fluid was obtained by inserting a tracheal tube and performing lung lavage (twice) with 0.8 ml of sterilized normal saline containing 2% bovine serum albumin. The collected lavage fluid was centrifuged at 500 × *g* at 4°C for 10 min. Then, the supernatants were harvested and stored at -80°C for subsequent cytokine measurements. Later, interleukin levels (INF-γ, IL-4, IL-5, IL-13, and IL-17A) in the BALF supernatant were analyzed by ELISA in accordance with the manufacturer’s instructions.

### Histopathological Evaluation of the Lung

Following BALF collection, lung tissue slices were fixed with 10% neutral-buffered formalin. Thin sections (3–4 μm) were the cut from the fixed tissue blocks and stained with hematoxylin and eosin (H&E). Histopathological assessment was then performed in a blind fashion using randomized sections and Image-Pro Plus software at a magnification of 100×. The severity of inflammatory cell infiltration in the airway was estimated using a five-point scoring system: 0, no cells; 1, a few cells; 2, a ring of cells, 1, cell layer deep; 3, a ring of cells, 2–4, cells deep; and 4, a ring of cells which was >4 cells deep (Jin et al., 2013).

### Flow Cytometric Analysis

Cells from the BALF were analyzed for the expression of Treg cells (Foxp3+CD4+CD25+) and Teff cells (Foxp3-CD25+CD4+) according to the manufacturer’s instructions. In brief, prepared cells were washed by centrifugation in flow cytometry staining buffer. Then, cells were stained with FITC-labeled anti-CD4 and APC-labeled anti-CD25 antibodies in staining buffer for 30 min at 4°C. Next, cells were fixed and permeabilized in a fixation/permeabilization solution for 30 min and subsequently stained with 0.5 μg of anti-rat Foxp3 PE. Finally, cells were resuspended in flow cytometry staining buffer and analyzed by flow cytometry using a FACS Calibur instrument and CellQuest software (BD Biosciences, Mountain View, CA, United States).

### Western Blot Analysis

Total protein was extracted from lung tissue using RIPA protein extraction reagent. An amount of 100 μg proteins were subjected to SDS–PAGE and transferred to PVDF membrane (Bio-Rad). Immunoblotting was then performed with phospho-p70S6K T389 (1:1000 dilution), p70 S6K (1:1000 dilution), phospho-S6 S235/236 (1:2000 dilution), phospho-Akt S473 (1:2000 dilution), Akt (1:2000 dilution), phospho-4E-BP1 S65 (1:1000 dilution), 4E-BP1 (1:1000 dilution), phospho-mTOR S2481 (1:1000 dilution) and mTOR (1:1000 dilution), GAPDH (1:2000 dilution), and secondary HRP-conjugated antibodies (1:10,000 dilution). Immunoreactivity was then detected by chemiluminescence according to the manufacturer’s instructions. Protein bands were then quantified using a calibrated Bio-Rad Image Lab densitometer and expressed as band intensity of phosphorylated proteins normalized to the relevant total proteins.

### Statistical Analysis

SPSS version 17 software (SPSS Inc., Chicago, IL, United States) was used to perform all statistical analysis. Data are expressed as means ± standard error of mean (SEM). The Shapiro–Wilk test was used to determine whether or not variables followed a normal distribution. The significance of the differences between groups was determined by one-way analysis of variance (ANOVA) or Kruskal–Wallis test, followed by *post hoc* Dunnett’s tests for comparisons between groups. A *p*-value of less than 0.05 was accepted as statistically significant.

## Results

### Molecular Characterization of MSJZT Using HPLC Chromatography

**FIGURE 2:**
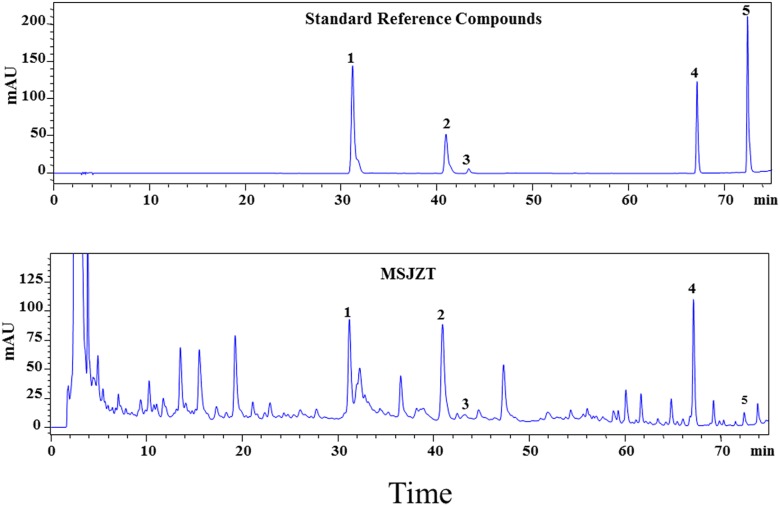
HPLC-UV chromatograms of MSJZT. Representative chromatograms of standard reference compounds and MSJZT. Five major components (1–5) were identified: calycosin-7-glucoside (1), hesperidin (2), lobetyolin (3), glycyrrhizic acid (4), and nobiletin (5).

To ensure the quality of the MSJZT used in our study, five major compounds were used as quality control standards for MSJZT. The HPLC chromatogram of MSJZT was achieved using a Platisil-C18 column and a mixture of acetonitrile and water with 0.1% phosphoric acid as the mobile phase. The HPLC chemical fingerprint of MSJZT is shown in Figure 2. The retention time of the five compounds calycosin-7-glucoside (1), hesperidin (2), lobetyolin (3), glycyrrhizic acid (4), and nobiletin (5) was 31.3, 41.0, 43.3, 67.3, and 72.6 min, respectively. The signal responses of compounds 3 and 5 were relatively low, thus, the ultraviolet spectrograms and HPLC chromatograms of related herbs in MSJZT were also provided, which supported that these compounds were indeed a part of the extract (Supplementary Figure S1).

### Oral Administration of MSJZT Suppressed OVA-Induced AHR

**FIGURE 3:**
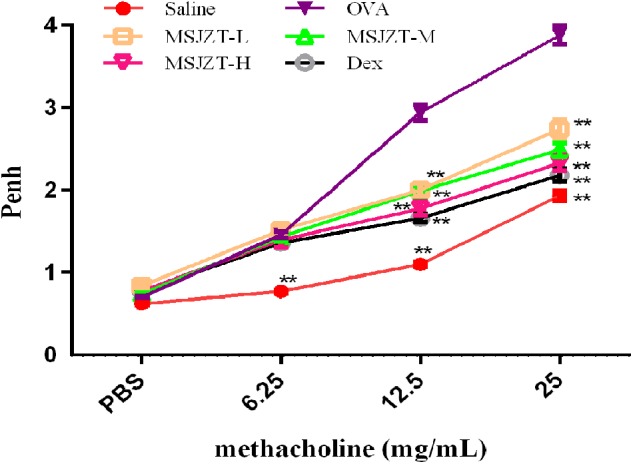
MSJZT suppressed Mch-induced AHR. Airway responsiveness to aerosolized Mch was evaluated by Buxco’s whole-body barometric plethysmography in awake, unrestrained mice. The mice were nebulized with PBS followed by increasing doses (6.25–25 mg/mL) of Mch. The Penh index of airway hyperreactivity was used as an indicator for changes in airway resistance. AHR was significantly increased after OVA re-exposure compared to saline controls. Treatment with MSJZT at 12.5, 25, and 50 g/kg and dexamethasone (Dex) (0.5 mg/kg) caused a marked decrease in Penh to Mch at 12.5 and 25 mg/ml compared to mice in the model group (*p* < 0.01). Data are expressed as means ± SEM (*n* = 10 in each group). ^∗∗^*P* < 0.01 versus OVA group.

Airway responsiveness to Mch was non-invasively measured by WBP in all mice in order to evaluate the effect of MSJZT on OVA-induced AHR. The Penh was measured as an indicator of bronchial responsiveness at baseline and after the sequential delivery of increasing concentrations of Mch (6.25–25 mg/ml). As shown in Figure 3, mice in the model group exhibited substantially enhanced AHR to Mch which was manifested by increased Penh compared with mice in the saline group (*p* < 0.01). Treatment with MSJZT at 12.5, 25, and 50 g/kg and Dex caused a marked reduction in Penh to Mch at 12.5 and 25 mg/ml compared to mice in the model group (*p* < 0.01). The effect of MSJZT on the suppression of AHR was similar to that of Dex.

### MSJZT Regulates Th2, and Th17 Cytokine Levels in BALF and IgE in Serum

**FIGURE 4:**
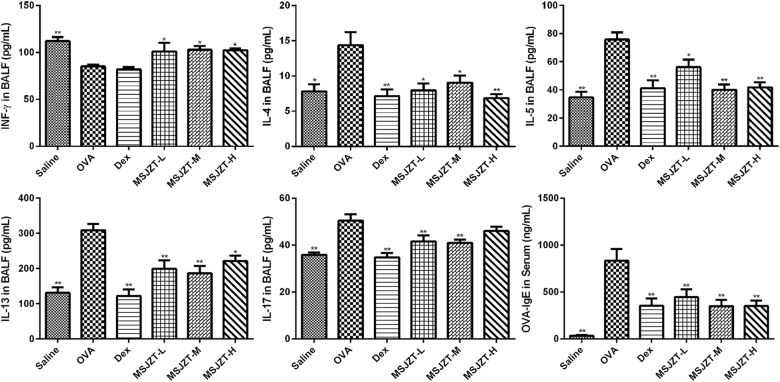
MSJZT inhibited inflammatory cytokine secretion and IgE. Secreted levels of INF-γ, IL-4, IL-5, IL-13, and IL-17 cytokines in BALF, and levels of antigen-specific IgE in serum, were measured by ELISA. Data are expressed as means ± SEM (*n* = 10 in each group). ^∗^*P* < 0.05 and ^∗∗^*P* < 0.01 *versus* the OVA group.

Airway inflammation in asthma is characterized by elevated levels of inflammatory cytokines. Therefore, in order to evaluate the effects of MSJZT treatment on inflammatory responses in asthmatic mice, we measured Th1, Th2, and Th17 cytokines in BALF. As shown in Figure 4, asthmatic mice exhibited markedly increased levels of Th2 (IL-4, IL-5, IL-13) and Th17 (IL-17A) cytokines, and reduced levels of Th1 (INF-γ) cytokine compared with mice in the saline group (*p* < 0.01). Following the oral administration of MSJZT or Dex, the levels of IL-4, IL-5, IL-13, and IL-17a dramatically reduced in the BALF (*p* < 0.05).

In addition, we measured antigen-specific IgE in serum to investigate the effects of MSJZT on allergic responses in asthma. IgE was significantly increased in serum after chronic OVA challenge (*p* < 0.01). However, there was an obvious reduction of IgE in serum after treatment with MSJZT or Dex (*p* < 0.01).

### Histopathological Examination of the Inhibitory Effect of MSJZT on Airway Inflammation

**FIGURE 5:**
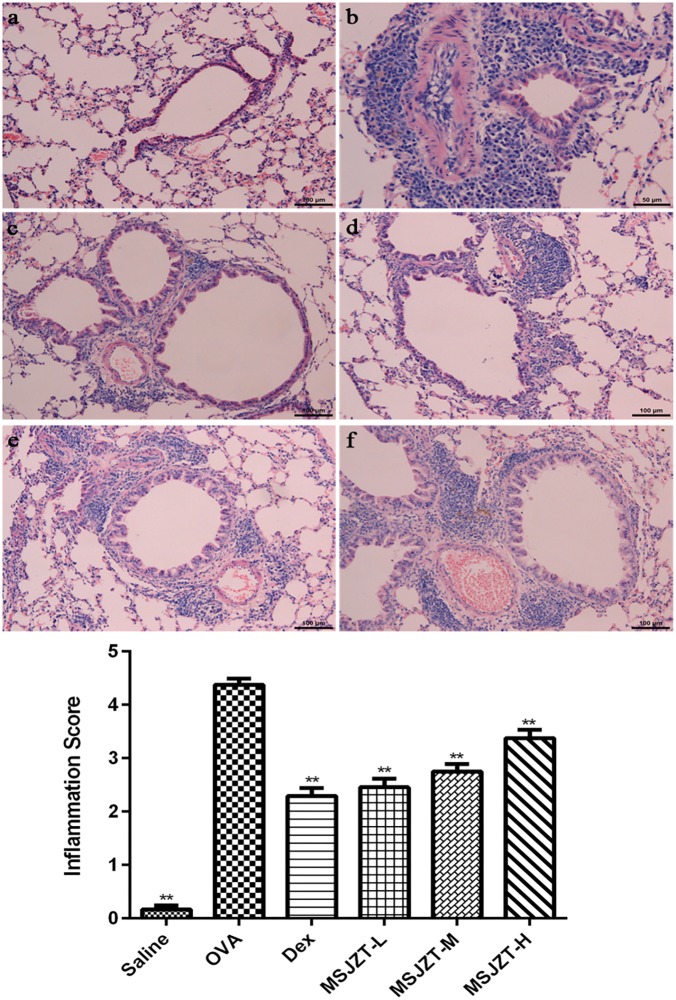
MSJZT attenuated airway inflammation in lung tissue. Lung tissue slices were fixed, embedded, sectioned at 3–4 μm, stained with hematoxylin and eosin, and then observed under a microscope (100×). The inflammation score represents the severity of inflammatory cell infiltration in the airway. Representative photomicrographs from each group (n = 10 per group) are shown as follows: **(a)** saline; **(b)** OVA; **(c)** dexamethasone (Dex) (0.5 mg/kg/day); **(d)** MSJZT-L (12.5 g/kg); **(e)** MSJZT-M (25 g/kg); and **(f)** MSJZT-L (50 g/kg). Data are expressed as means ± SEM. ^∗∗^*P* < 0.01 versus the OVA group.

Lung tissue was examined after H&E staining in order to confirm the inhibitory effect of MSJZT on airway inflammation. As shown in Figure 5, tissue specimens showed severe infiltration of inflammatory cells in the perivascular and peribronchiolar areas of the model group compared to the saline group (*p* < 0.01). However, the oral administration of MSJZT and Dex dramatically attenuated the inflammatory infiltrate around the airways and blood vessels. Finally, there was no significant difference among MSJZT- and Dex-treated groups (*p* > 0.05).

### MSJZT Causes a Depletion of CD4+CD25+Foxp3-Teff Cells in BALF

**FIGURE 6:**
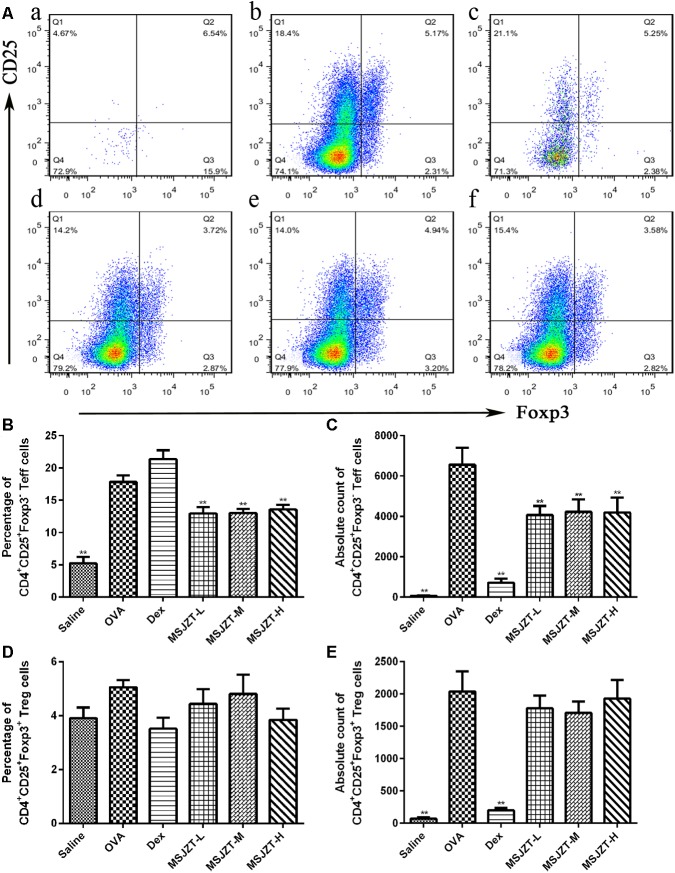
MSJZT inhibited levels of CD4^+^ CD25^+^ Foxp3^-^ Teff cells in BALF. **(A)** Representative plots from a single mouse in each group. Numbers inside the histograms indicate the percentage of cells. Representative images are shown for each group: **(a)** saline, **(b)** OVA, **(c)** dexamethasone (Dex) (0.5 mg/kg/day), **(d)** MSJZT-L (12.5 g/kg), **(e)** MSJZT-M (25 g/kg), and **(f)** MSJZT-L (50 g/kg). **(B,C)** Percentage and absolute number of CD4^+^ CD25^+^ Foxp3^-^ Teff cells. **(D,E)** Percentage and absolute number of CD4^+^ CD25^+^ Foxp3^+^ Treg cells. Data are expressed as means ± SEM (*n* = 6 in each group). ^∗∗^*P* < 0.01 *versu*s the OVA group.

The mTOR signaling pathway plays a significant role in the growth and proliferation of CD4+ T cells. Foxp3 is an invaluable marker of Treg cells (Foxp3+CD25+CD4+) and can distinguish these cells from their thymic progenitors and activated Teff cells (Foxp3-CD25+CD4+). Therefore, we focused on the effects of MSJZT on these cells. When Teff and Treg cells were assessed as a proportion of the total population of CD4+ T cells, MSJZT clearly suppressed the proportion of OVA-induced Teff cells but not Treg cells. Total Teff cell numbers were remarkably increased in OVA-exposed mice compared to saline controls and were completely suppressed by treatment with MSJZT and dexamethasone. However, MSJZT had limited effects on the total number of Treg cells, which was significantly reduced by Dex (Figure 6). The full gating strategy was also provided followed for characterization of the cell populations present in the BALF (Supplementary Figure S2).

### MSJZT Treatment Inhibited the mTORC1 Pathway, but Not the mTORC2 Pathway

**FIGURE 7:**
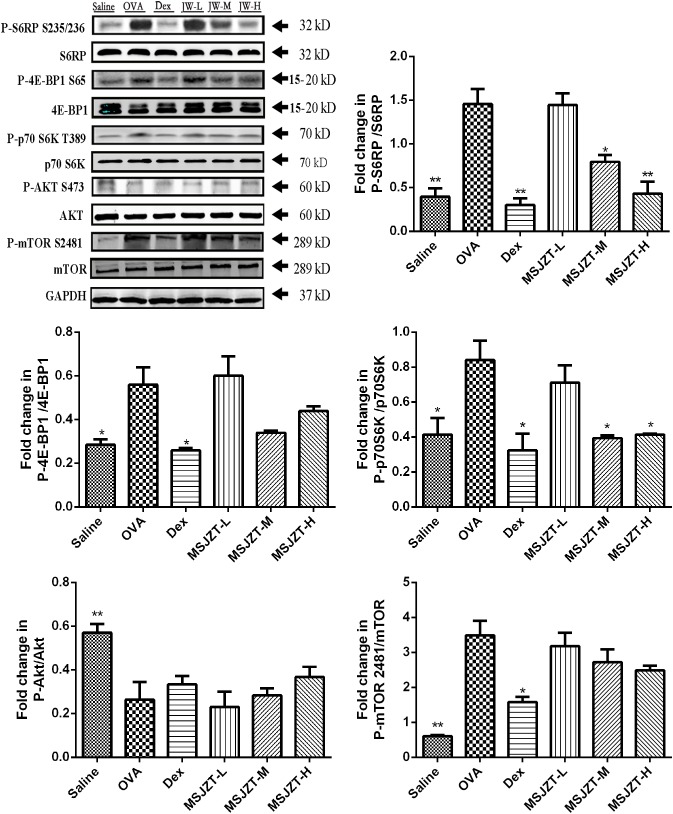
MSJZT treatment inhibited the mTORC1 pathway, but not the mTORC2 pathway in lung homogenates. Western blot analysis was performed to investigate mTORC1 and mTORC2 activities and involved investigating the phosphorylation of their substrates, S6RP, p70S6K, 4E-BP1, Akt, and mTOR 2481, respectively. The expression of these proteins was quantified and represented as band intensity of phosphorylated proteins normalized to the relevant total proteins. The amount of loaded material was 100 μg. Data are expressed as means ± SEM (*n* = 6 in each group). ^∗^*P* < 0.05 and ^∗∗^*P* < 0.01 *versus* the OVA group.

mTOR signaling has previously been shown to play an important role in the proliferation of T cells. To determine whether the effects of MSJZT on Teff cells in asthmatic mice were associated with the mTOR pathway, we investigated mTORC1 and mTORC2 activity by determining the phosphorylation of their substrates S6RP, p70S6K, 4E-BP1, Akt, and mTOR 2481. OVA exposure significantly augmented S6RP, p70S6K, 4E-BP1, and mTOR 2481 phosphorylation compared to saline controls. Furthermore, phosphorylation of the mTORC1 substrates, S6RP and p70S6K, but not 4E-BP1, was significantly inhibited by MSJZT treatment at median and high doses (25 and 50 g/kg). However, phosphorylation of the mTORC2 substrates, Akt and mTOR 2481, was not suppressed by MSJZT treatment. When taken together, these findings can be seen to indicate that MSJZT blocked mTORC1, but not mTORC2 (Figure 7).

## Discussion

Modified Si–Jun–Zi–Tang has been widely used for centuries in China to prevent asthma attacks. However, the mechanisms underlying the action of MSJZT remain unclear and its effects on airway inflammation remain to be fully assessed. To further understand the anti-asthmatic mechanisms of MSJZT, we used a chronic mouse model of asthma in the present study. Consistent with previous reports, OVA sensitization and repeated challenges led to significant AHR, inflammatory cell infiltrates, higher antigen-specific IgE levels, and increased Th2–Th17 responses. In this study, the effect of MSJZT on airway responsiveness was monitored using the Penh, which demonstrated that MSJZT dramatically inhibited AHR in asthmatic mice. Our results also showed that MSJZT reduced bronchiolar and perivascular inflammatory cell infiltrates, thus reducing airway inflammation. Aberrant production of the Th2 and Th17 cytokines, IL-4, IL-5, IL-13, and IL-17, mediate the induction of the IgE isotype switch, which has long been associated with the pathogenesis of asthma (Gandhi et al., 2016). In the present study, we found that MSJZT suppressed levels of Th2 and TH17 cytokines including IL-4, IL-5, IL-13, and IL-17a, consistent with reduced levels of OVA-specific IgE in serum. It appears that inhibition of Th2–TH17 response may contribute to the anti-asthmatic effects of MSJZT.

We further investigated whether the suppressive effect of MSJZT on Th2 and Th17 responses was partly due to an increased number of Treg cells or inhibition of Teff cell function in MSJZT-treated mice. Foxp3 is currently the most specific marker in terms of distinguishing Treg cells (Foxp3+CD25+CD4+) from activated Teff cells (Foxp3-CD25+CD4+). Treg cells have been recently reported to suppress Teff cell-mediated immune responses. The *in vivo* transfer of CD4+CD25+Treg cells was previously shown to inhibit AHR and Th2-mediated airway inflammation in a mouse model of asthma (Kearley et al., 2005). However, it was unknown whether Treg cells were involved in the anti-asthmatic effects of MSJZT. Our present study showed that MSJZT treatment does not increase the proportion or absolute number of Treg cells in the total CD4+T cell sub-population. These results indicated that anti-asthmatic action of MSJZT was not mediated through the induction of Treg cells. However, our results revealed that treatment of allergic mice with MSJZT significantly reduced the number and percentage of Teff cells (Foxp3-CD25+CD4+) in total CD4+T cell subpopulation. Previous studies have demonstrated that the development of CD4+Teff cells, such as Th2 and Th17 cells, strongly contribute to airway inflammation, mucus production, AHR, and airway remodeling concomitant with high circulating levels of IgE in patients with allergic asthma (Zuska-Prot and Maslanka, 2017). Collectively, our findings suggest that MSJZT treatment attenuated Th2 and Th17 cell responses to limit allergen-induced immunopathology, at least in part, by inhibiting the number and proportion of Teff cells (Foxp3-CD25+CD4+).

The mTOR signaling, including two distinct signaling complexes, mTORC1 and mTORC2, is a central regulator of cell metabolism, growth, proliferation, and survival and is known to play a significant role in the pathogenesis of asthma (Laplante and Sabatini, 2012). A previous study showed that mTOR signaling was significantly activated in patients with asthma onset (Zhang et al., 2017), and that the inhibition of mTOR by rapamycin remarkably attenuated airway hyper-reactivity and inflammation in asthmatic mice (Mushaben et al., 2011). More recently, the differentiation and activation of CD4+ T cells has been shown to depend upon the mTOR signaling pathway (Angela et al., 2016). The mTORC1 pathway regulates the polarization and function of Th2 and Th17 cells, and Th2 cell proliferation can be inhibited via an mTORC1/S6 kinase-1-dependent pathway (Yang et al., 2013; Ren et al., 2016; Yin et al., 2017). In another study, phospho-S2481 on mTOR was reported to be a biomarker for the activation of mTORC2 signaling (Copp et al., 2009), and mTORC2 was shown to control Th9 polarization in allergic airway inflammation (Chen et al., 2017). Thus, we investigated whether the inhibitory effects on Teff cells were associated with mTORC1/2 signaling. In the present study, the activities of mTORC1 and mTORC2 were assessed by investigating the phosphorylation of their downstream targets, S6RP, p70S6K, 4E-BP1, Akt, and mTOR 2481. We confirmed that mTOR signaling was dramatically activated in our asthma model, as phosphorylation of the mTOR downstream targets, S6RP, p70S6K, 4E-BP1, and mTOR 2481, was dramatically increased. MSJZT markedly suppressed OVA-induced increases in the levels of phosphorylated p70S6K and S6RP, although the phosphorylation of Akt and mTOR 2481 did not change significantly. These results indicated that MSJZT could effectively block mTORC1 without affecting mTORC2. Interestingly, MSJZT inhibited mTORC1 by affecting the phosphorylation status of S6K1/S6RP but not 4EBP1. One explanation for this observation might be that S6K1 and 4E-BP1 were independently regulated, and that the phosphorylation of 4E-BP1 could be regulated directly or indirectly by PI3K but not via mTORC1 (Nawroth et al., 2011).

Modified Si–Jun–Zi–Tang is a traditional Chinese formula composed of seven different herbs. Some herb compositions of MSJZT such as *A. membranaceus* (*Fisch.*) *Bunge, Citri Reticulatae Pericarpium, G. glabra L*., or *P. pedatisecta Schott* have been demonstrated to possess anti-asthmatic effects (Ok et al., 2009; Shi et al., 2009; Jin et al., 2013; Li et al., 2014). However, the active component of MSJZT remains unknown. In the present study, HPLC analysis identified five chemicals in MSJZT (calycosin-7-glucoside, hesperidin, lobetyolin, glycyrrhizic acid, and nobiletin); previous studies have indicated that these compounds significantly attenuate allergic airway inflammation by inhibiting the Th2 cell response in asthmatic mice (Wu et al., 2006; Kim et al., 2011; Ma et al., 2013; Wei et al., 2015). Furthermore, hesperidin, glycyrrhizic acid, and nobiletin are known to exert inhibitory effects on mTOR signaling (Saiprasad et al., 2014; He et al., 2015; Qu et al., 2018) and may represent the active components of MSJZT; consequently, these compounds may prevent airway inflammation via such effects on mTOR. Further studies are now needed to elucidate these hypotheses.

One limitation of our study was that the amount of compounds identified was relatively low. In fact, we also perform HPLC analysis on the other chemicals such as pachymic acid, atractylenolide II, and atractylenolide I. We have tried many times and set the optimum analysis condition to get signal responses of compounds as much as possible. However, we did not found the signal response of the above chemicals in MSJZT, which might due to the low content of these compounds in MSJZT or the limitation of the HPLC analysis technique. Previous studies have identified more compounds in a similar decoction namely Si–Jun–Zi–Tang such as Ferulic acid, Liquiritin, Benzoic acid, etc. using the methods of UPLC – with photodiode array detector or HPLC–ESI–MS (Zhang et al., 2011; Huang et al., 2015). Thus, further studies using more advanced techniques are still needed to identify more critical components in MSJZT.

Modified Si–Jun–Zi–Tang is a commonly safe Chinese herbal prescription. We acknowledge that the dosages of MSJZT used in our study were relatively high. However, no significant side-effects such as body-weight loss or pathological change in lung tissues were observed. A recent study also used a high level dosage of a similar decoction with 30.0 g/kg for rats which is equal to about 45 g/kg for mice, whereas no distinct toxicity and side effects were observed (Yu et al., 2016). However, toxicity or side effects of MSJZT are still not fully understood, and further studies are needed to assess toxicity of MSJZT.

## Conclusion

This study indicated that the administration of MJZT during a phase of remission could suppress airway inflammation by inhibiting Teff cells (Foxp3-CD25+CD4+) in a mouse model of chronic asthma. The mechanism involved appears to depend on the suppression of the mTORC1 signaling pathway.

## Ethics Statement

This study was carried out in accordance with the recommendations of “Zhejiang Regulation for the Administration of Laboratory Animals.” The protocol was approved by the “Institutional Animal Care and Use Committee of Zhejiang Chinese Medical University (permit number: ZSLL-2016-37; February 2016).

## Author Contributions

HJ was responsible for the basic idea and wrote the draft manuscript. CC, BL, WJ, and JX carried out the experiments and collected the data. SM and LW were responsible for overall supervision of manuscript writing and editing. All authors read and approved the final manuscript.

## Conflict of Interest Statement

The authors declare that the research was conducted in the absence of any commercial or financial relationships that could be construed as a potential conflict of interest.

## Supplementary Material

FIGURE S1 The ultraviolet spectrograms and HPLC chromatograms. **(A)** The ultraviolet spectrograms of MSJZT and lobetyolin standard compound. **(B)** The ultraviolet spectrograms of MSJZT and nobiletin standard compound. **(C)** HPLC chromatograms of MSJZT, negative control (similar to MSJZT but without codonopsis pilosula), the single herb of codonopsis pilosula, and the standard compound of lobetyolin. **(D)** HPLC chromatograms of MSJZT, negative control (similar to MSJZT but without *Citri Reticulatae Pericarpium*), the single herb of *Citri Reticulatae Pericarpium* and the standard compound of nobiletin.

FIGURE S2 Flow cytometric characteristics and the full gating strategy. **(A)** Distinction of lymphocytes, eosinophils, lymphocytes, and macrophages based on the morphological flow cytometric parameters FSC and SSC. **(B)** Lymphocytes subset in BALF cells were gated as FSClo/SSClo, and then FITC-CD4-positive lymphocytes were selected. Finally, Teff and Treg cells were identified as CD4+CD25+Foxp3- and CD4+CD25+Foxp3+ cells within the gate of CD4-positive lymphocytes.

## Abbreviations

AHR: airway hyper-responsiveness
BALF: bronchoalveolar lavage fluid
Dex: dexamethasone
GCs: glucocorticoids
HPLC: high-performance liquid chromatography
IgE: immunoglobulin E
Mch: methacholine
MSJZT: modified Si–Jun–Zi–Tang
Penh: enhanced pause
OVA: ovalbumin
Teff: T effector
Treg: T regulatory
WBP: whole-body plethysmography
