# Supplementary figures and images for: Modified Si–Jun–Zi–Tang Attenuates Airway Inflammation in a Murine Model of Chronic Asthma by Inhibiting Teff Cells via the mTORC1 Pathway
Source: Front Pharmacol. 2019 Feb 27;10:161. doi: 10.3389/fphar.2019.00161 (PMC6400882; doi:10.3389/fphar.2019.00161)

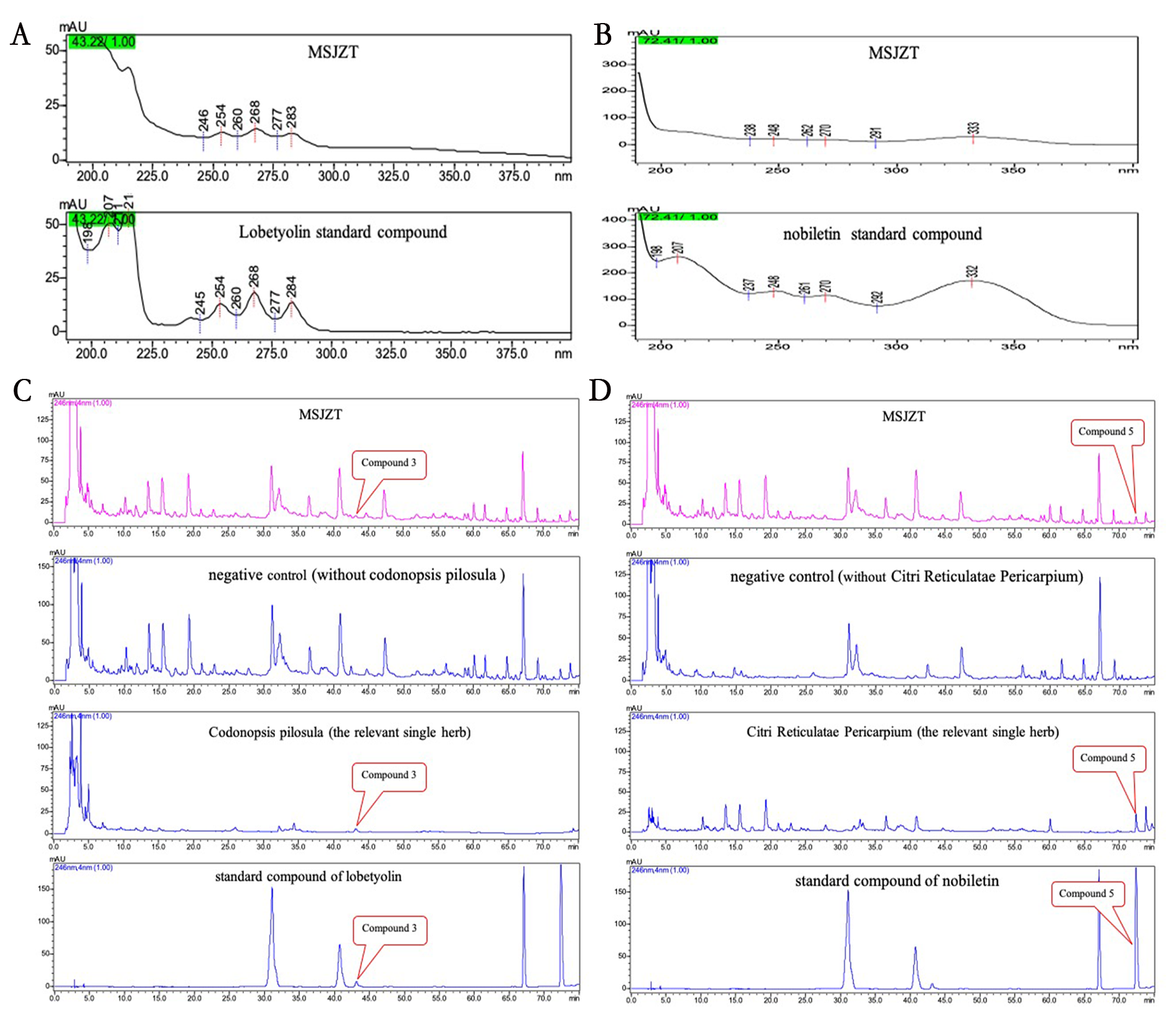

Supplement: FIGURE S1 — The ultraviolet spectrograms and HPLC chromatograms. (A) The ultraviolet spectrograms of MSJZT and lobetyolin standard compound. (B) The ultraviolet spectrograms of MSJZT and nobiletin standard compound. (C) HPLC chromatograms of MSJZT, negative control (similar to MSJZT but without codonopsis pilosula), the single herb of codonopsis pilosula, and the standard compound of lobetyolin. (D) HPLC chromatograms of MSJZT, negative control (similar to MSJZT but without Citri Reticulatae Pericarpium), the single herb of Citri Reticulatae Pericarpium and the standard compound of nobiletin. [file Image_1.TIF]

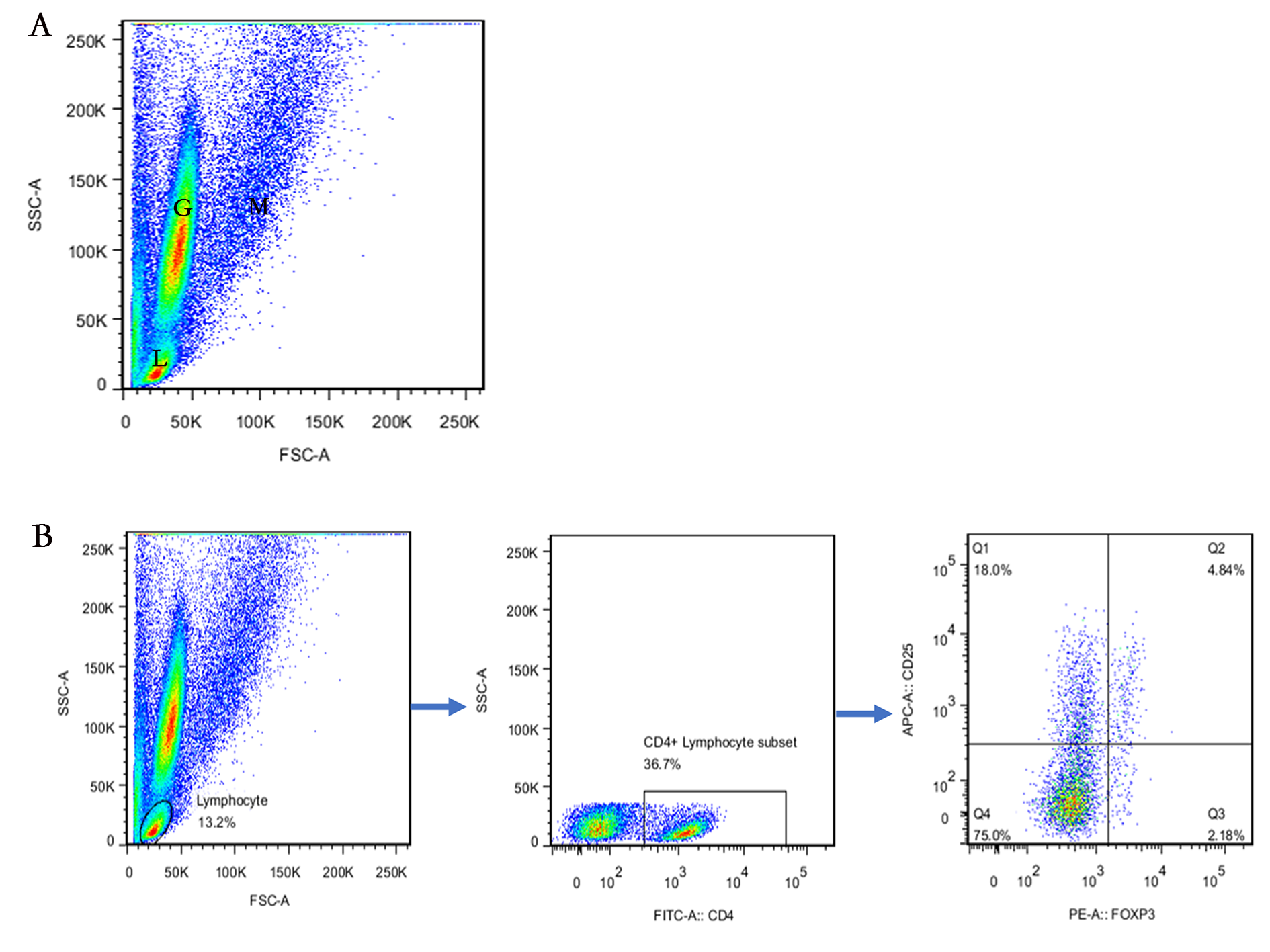

Supplement: FIGURE S2 — Flow cytometric characteristics and the full gating strategy. (A) Distinction of lymphocytes, eosinophils, lymphocytes, and macrophages based on the morphological flow cytometric parameters FSC and SSC. (B) Lymphocytes subset in BALF cells were gated as FSClo/SSClo, and then FITC-CD4-positive lymphocytes were selected. Finally, Teff and Treg cells were identified as CD4+CD25+Foxp3- and CD4+CD25+Foxp3+ cells within the gate of CD4-positive lymphocytes. [file Image_2.TIF]
